# Lumbar Disc Degeneration and Vertebral Fracture at the Thoracolumbar Junction Are Risk Factors for Chronic Low Back Pain With Disability: Seven Years’ Follow-Up of the Wakayama Spine Study

**DOI:** 10.7759/cureus.84291

**Published:** 2025-05-17

**Authors:** Naomi Iwane, Hiroshi Hashizume, Shizumasa Murata, Kanae Mure, Hiroyuki Oka, Toshiko Iidaka, Masatoshi Teraguchi, Keiji Nagata, Yuyu Ishimoto, Masanari Takami, Shunji Tsutsui, Hiroshi Iwasaki, Sakae Tanaka, Hiroshi Yamada, Noriko Yoshimura

**Affiliations:** 1 School of Health and Nursing Science, Wakayama Medical University, Wakayama, JPN; 2 Department of Orthopaedic Surgery, Wakayama Medical University, Wakayama, JPN; 3 Department of Public Health, Wakayama Medical University, Wakayama, JPN; 4 Division of Musculoskeletal AI System Development, Faculty of Medicine, The University of Tokyo, Tokyo, JPN; 5 Department of Preventive Medicine for Locomotive Organ Disorders, 22nd Century Medical and Research Center, The University of Tokyo, Tokyo, JPN; 6 Department of Orthopaedic Surgery, Faculty of Medicine, The University of Tokyo, Tokyo, JPN

**Keywords:** chronic low back pain, longitudinal study, lumbar disc degeneration, lumbar spinal stenosis, vertebral fracture

## Abstract

Introduction

Low back pain (LBP) is the leading cause of disability worldwide, with its burden increasing in aging societies such as Japan. Although degenerative spinal changes like lumbar disc degeneration (DD), vertebral fractures, and lumbar spinal stenosis (LSS) are frequently identified on MRI, their combined longitudinal impact on disabling chronic low back pain (DCLBP) remains unclear. The aim of this study was to identify baseline MRI-detected lumbar spinal changes that independently predict disabling chronic low back pain in a general Japanese population.

Methods

This population-based longitudinal study included 663 community-dwelling Japanese adults from the Wakayama Spine Study, a sub-cohort of the nationwide Research on Osteoarthritis/Osteoporosis Against Disability (ROAD) study. Baseline whole-spine MRI and clinical assessments were conducted between 2008 and 2009, with a seven-year follow-up from 2015 to 2016. MRI findings included Pfirrmann-graded lumbar DD, Genant-graded vertebral fractures at T11-L1, and Suri-graded LSS. The primary outcome was DCLBP, defined as LBP lasting more than three months and an Oswestry Disability Index (ODI) score ≥21%. Multivariate logistic regression was used to identify independent predictors of DCLBP.

Results

Of 653 participants who completed follow-up with valid ODI responses, 91 (13.9%) had DCLBP. Older age (OR: 1.07 per year, p < 0.0001), female sex (OR: 3.69, p < 0.0001), higher BMI (OR: 1.11 per kg/m², p < 0.0001), greater vertebral fracture burden (OR: 1.32 per grade point, p = 0.0024), and more severe lumbar DD (OR: 1.14 per grade point, p = 0.0305) were independently associated with DCLBP.

Conclusion

Lumbar DD, vertebral fractures at T11-L1, and LSS are independent risk factors for disabling chronic LBP in the general population. These findings underscore the importance of comprehensive MRI-based spinal assessment in identifying high-risk individuals for early intervention in aging societies.

## Introduction

Low back pain (LBP) is the leading cause of disability worldwide, affecting approximately 619 million people in 2020 and projected to increase further with global population aging [1]. In Japan, where more than 28% of the population is aged 65 years or older, the burden of chronic LBP is especially pronounced, contributing significantly to limitations in activities of daily living (ADLs) and reduced quality of life [2].

With the widespread availability of magnetic resonance imaging (MRI), spinal degenerative changes are increasingly detected in both clinical and research settings. Notably, many of these findings are observed even in asymptomatic individuals, complicating efforts to establish their clinical relevance [3]. Among the most frequently identified MRI abnormalities are lumbar disc degeneration (DD), vertebral fractures, particularly in the thoracolumbar junction due to osteoporosis, and lumbar spinal stenosis (LSS). These structural changes have all been associated with chronic LBP in both Western and Asian populations [4-6]. For example, Pfirrmann grade 4 or 5 disc degeneration has been linked to pain-related functional impairment, while morphometric vertebral fractures are associated with postural instability and mechanical pain [7]. LSS, when assessed on axial MRI, can lead to central canal narrowing, neural compression, and neurogenic claudication [8].

However, these degenerative abnormalities often coexist within the same individual, particularly in older adults. This anatomical overlap complicates the interpretation of their individual and combined contributions to chronic disabling pain [9]. Moreover, most previous studies have examined these findings in isolation, typically in cross-sectional or hospital-based settings, and often without adjustment for coexisting spinal pathologies. As a result, there remains a critical gap in the literature regarding the longitudinal interplay of multiple MRI-detected spinal abnormalities and their association with long-term functional disability.

Another methodological shortcoming in prior studies is the underutilization of validated tools to assess the real-world impact of LBP. While many investigations focus on pain intensity or radiologic severity, relatively few evaluate disability using function-oriented measures such as the Oswestry Disability Index (ODI), which quantifies disability across multiple dimensions of daily life and provides clinically meaningful thresholds [10]. In addition, findings from hospital-based samples may not generalize to community-dwelling populations, who represent the majority of older adults at risk for developing disabling LBP.

To our knowledge, no previous large-scale, population-based, longitudinal study has simultaneously evaluated lumbar DD, vertebral fractures, and LSS using MRI as predictors of disabling chronic low back pain (DCLBP) over an extended follow-up period. This limitation is particularly notable in non-Western populations, including Japan, where demographic aging is more advanced and culturally specific risk profiles may exist.

To address this gap, we conducted a seven-year longitudinal study utilizing data from the Wakayama Spine Study (WSS), a sub-cohort of the nationwide Research on Osteoarthritis/Osteoporosis Against Disability (ROAD) study. This cohort comprises community-dwelling Japanese adults with comprehensive MRI and questionnaire-based data. We aimed to determine which baseline degenerative changes - lumbar disc degeneration, vertebral fractures at T11-L1, and LSS - independently predict the development of DCLBP, defined as chronic LBP lasting more than three months with an ODI score ≥21%.

The objective of this longitudinal, population-based study was to evaluate whether lumbar disc degeneration, vertebral fractures at the thoracolumbar junction (T11-L1), and LSS, as assessed by MRI, independently predict the development of DCLBP after seven years of follow-up in community-dwelling Japanese adults.

This study is unique in its design, scale, and outcome measures. It integrates high-resolution MRI of the lumbar spine, validated disability assessment, and a long follow-up duration in a general population. By simultaneously evaluating multiple structural abnormalities while adjusting for key demographic and health-related confounders, our findings aim to clarify the cumulative and individual contributions of spinal degeneration to real-life disability. The results have direct implications for the early identification of at-risk individuals and the development of targeted prevention strategies in aging societies worldwide.

## Materials and methods

This was a population-based, longitudinal cohort study using prospectively collected data from the Wakayama Spine Study, a sub-cohort of the nationwide ROAD study in Japan. The study procedures included baseline assessments (MRI, radiographs, and questionnaires) conducted between 2008 and 2009, and follow-up assessments (chronic low back pain and ODI) conducted between 2015 and 2016.

Ethical statement

All procedures involving human participants in this study were conducted in accordance with the ethical standards of the institutional and national research committees and with the 1964 Helsinki Declaration and its later amendments or comparable ethical standards. The study protocol was approved by the ethics committees of the University of Tokyo (approval nos. 1326). Written informed consent was obtained from all participants prior to enrollment.

Participants

This study was conducted as part of the WSS, a population-based cohort study focusing on age-related degenerative changes of the spine in community-dwelling residents. The WSS is a sub-cohort of the nationwide ROAD study, a large-scale, multicenter prospective cohort investigating musculoskeletal health in the Japanese population. Detailed protocols of the ROAD study have been reported elsewhere. Participants were recruited from two communities in Wakayama Prefecture, Hidakagawa (mountainous area) and Taiji (coastal area), through resident registries [11,12].

Among 1607 invited residents, a total of 1011 individuals provided written informed consent and participated in the baseline WSS from 2008 to 2009. Each participant underwent whole-spine MRI on the same day as clinical examination using a mobile MRI unit (Excelart 1.5 T, Toshiba, Tokyo, Japan). After excluding two individuals with pacemakers, 1009 subjects (335 men and 674 women; mean age, 66.3 years; age range, 21-97 years) were included in the baseline cohort.

**Figure 1:**
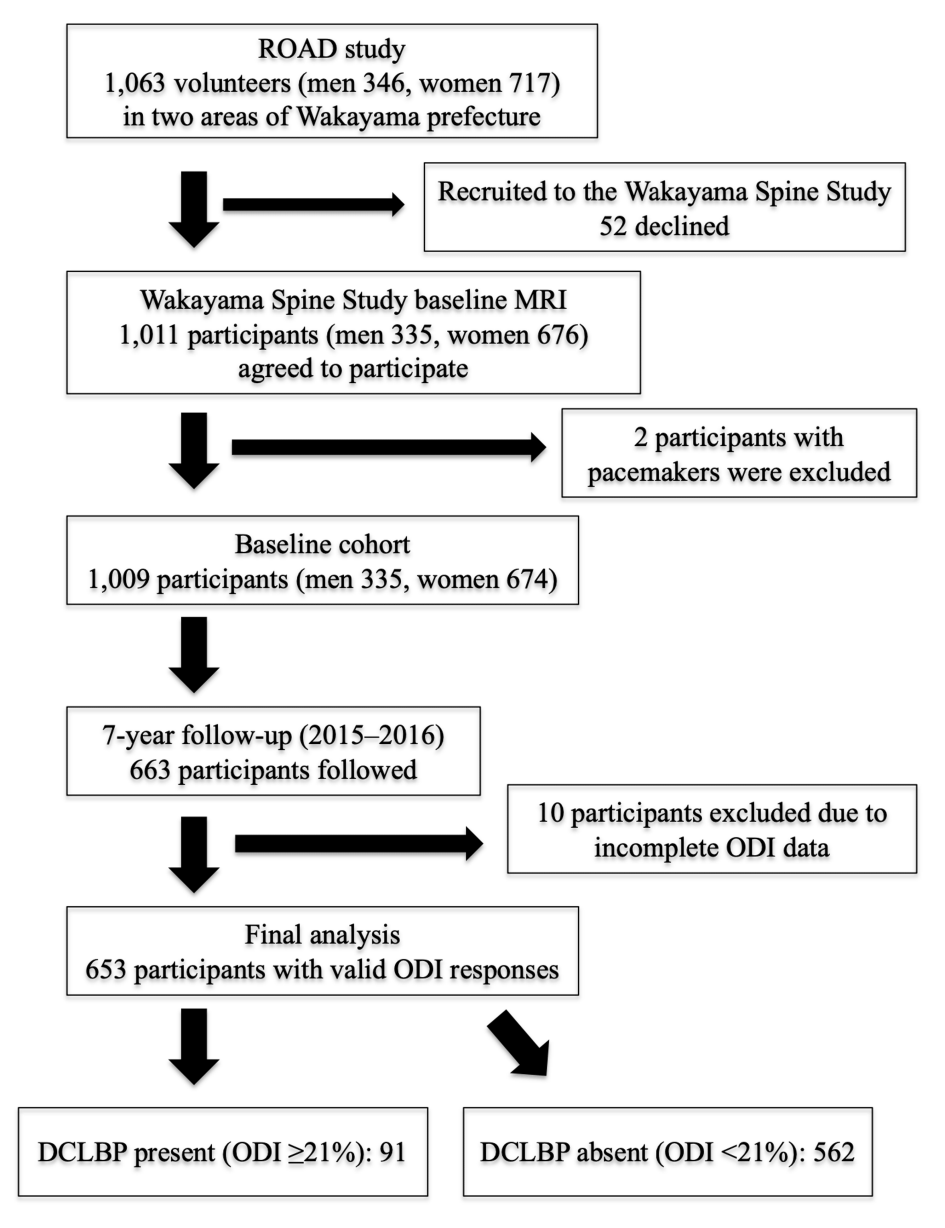
Participant flow diagram from recruitment to final analysis. A total of 1,063 volunteers from two regions of Wakayama Prefecture were initially recruited from the Research on Osteoarthritis/Osteoporosis Against Disability (ROAD) study. After exclusion of 52 individuals who declined participation, 1,011 participants consented to undergo baseline full-spine MRI as part of the Wakayama Spine Study. Two participants were excluded due to MRI contraindications (presence of pacemakers), resulting in 1,009 eligible individuals at baseline. Among them, 663 completed the seven-year follow-up survey, and 653 had valid responses for the Oswestry Disability Index (ODI), forming the final analytic cohort. Of these, 91 participants (13.9%) were classified as having disabling chronic low back pain (DCLBP), defined as chronic low back pain lasting three or more months with an ODI score ≥21%. Values are presented as number and percentage (N, %). Statistical significance was set at p<0.05.

In the third survey of the Wakayama Spine Study, conducted between 2015 and 2016, longitudinal follow-up was completed for 663 participants (219 men and 444 women; mean age at baseline, 62.1 ± 13.0 years). These 663 individuals formed the final study population for the current analysis, in which the presence of DCLBP was assessed after seven years of follow-up (Figure 1).

Assessment and outcome measures

At baseline (2008-2009), demographic and lifestyle information was collected from each participant via a structured, interviewer-administered questionnaire consisting of 400 items. This comprehensive survey covered occupation, smoking habits, alcohol consumption, family and medical history, physical activity, reproductive variables, and health-related quality of life (HRQoL). Smoking status was categorized as either current smoker (any tobacco use regardless of amount) or non-smoker (never or former smoker). Alcohol use was similarly classified as current habitual drinker or non-drinker. Anthropometric measurements, including height and weight, were obtained by trained staff, and body mass index (BMI) was calculated as weight (kg) divided by height squared (m²).

The mental component summary (MCS) score was calculated using the validated Japanese version of the Short Form-8 Health Survey (SF-8), a widely used instrument for assessing HRQoL in epidemiological research [13].

MRI of the entire spine was performed on the same day as the clinical examination using a 1.5-T mobile MRI unit (Excelart; Toshiba). Lumbar disc degeneration (L1/2-L5/S1) was graded using Pfirrmann’s classification (grades 1-5) on sagittal T2-weighted images, and the total degeneration score was calculated by summing the grades across all lumbar levels. Prevalent vertebral fractures at the thoracolumbar junction (T11-L1) were assessed using Genant’s semi-quantitative (SQ) method (grades 0-3), and the total fracture burden was defined as the sum of the SQ grades at these three levels. LSS was evaluated using axial MRI and classified at each level according to the Suri grading system (grades 0-3). Participants with grade 3 stenosis at any lumbar level were categorized as having “severe” LSS.

At the seven-year follow-up (2015-2016), participants were asked whether they had experienced low back pain lasting for more than three consecutive months. Those who responded affirmatively were classified as having chronic low back pain (CLBP). The degree of disability associated with CLBP was assessed using the validated Japanese version of the ODI. DCLBP was defined as the coexistence of CLBP and an ODI score ≥21%.

Assessment of lumbar disc degeneration

Lumbar disc degeneration (L1/2 to L5/S1) was evaluated using sagittal T2-weighted MRI images. An experienced board-certified orthopaedic surgeon (MT), blinded to participant background, assessed each level using the Pfirrmann classification (grades 1 to 5) [14]. Grades 4 and 5 were considered indicative of advanced degeneration. Grade 4 was defined by an inhomogeneous structure with intermediate to hypointense signal intensity relative to cerebrospinal fluid, whereas grade 5 was characterized by a black, inhomogeneous signal and collapsed disc space.

This method has been shown to correlate with both morphological and biochemical degeneration, including loss of water and proteoglycan content [15]. The grading system was selected for its reliability and reproducibility in evaluating disc signal intensity and height [16]. The total degeneration score was calculated by summing Pfirrmann grades from L1/2 to L5/S1. Inter- and intra-observer reliability, as determined by kappa statistics, were excellent (κ = 0.94 for both) [16].

Assessment of vertebral fractures

Thoracolumbar vertebral fractures (T11-L1) were assessed using the Genant SQ method [17], in accordance with the 2015 Official Positions of the International Society for Clinical Densitometry (ISCD) [18]. Lateral stand-up radiographs were obtained using standardized positioning protocols by licensed radiologic technologists [19]. Radiographs encompassed the region from the cervical vertebrae (C2) to the proximal femur. Participants stood upright with hips and knees fully extended, and arms flexed with hands placed on shoulder-height supports.

A spine surgeon (CH) evaluated vertebrae from T4 to the sacrum using the SQ method, grading each vertebra from 0 (normal) to 3 (severe deformity). Vertebrae with insufficient image quality were excluded. In this study, a prevalent vertebral fracture was defined as SQ grade ≥2 at T11, T12, or L1, given that grade 1 fractures are considered of limited clinical relevance. Fractures at the thoracolumbar junction (T11-L1) were specifically analyzed because this region represents a biomechanical transition zone between the rigid thoracic spine and the mobile lumbar spine, and is highly susceptible to osteoporotic fractures that impact sagittal alignment and functional outcomes.

To assess reliability, intra- and inter-observer agreement were evaluated using 50 randomly selected radiographs. The same evaluator (CH) reassessed the images after a two-week interval, while a second orthopaedist (TI), blinded to the initial scores, independently evaluated them. Kappa values were 0.70 (intra-observer) and 0.62 (inter-observer), with corresponding agreement rates of 98.1% and 98.0%, respectively [20].

Assessment of lumbar spinal stenosis

LSS was assessed on axial T2-weighted MRI at each lumbar intervertebral level. Although no consensus exists regarding a standardized MRI-based definition of LSS [21,22], this study adopted the qualitative grading system described by Suri et al. [8], which has also been applied in a Japanese population-based study [6].

A spine surgeon (YI), blinded to clinical information, graded the most stenotic slice at each level as follows: Grade 0: No narrowing; Grade 1 (Mild): ≤1/3 narrowing of the central canal; Grade 2 (Moderate): >1/3 to ≤2/3 narrowing; Grade 3 (Severe): >2/3 narrowing.

Participants were defined as having severe LSS if grade 3 stenosis was identified at any level. To evaluate reliability, intra-observer agreement was assessed by re-evaluating 50 randomly selected scans after a one-month interval, yielding a kappa of 0.82 (95% CI: 0.77-0.86). Inter-observer agreement, measured by comparison with a second experienced spine surgeon (KN), was 0.77 (95% CI: 0.73-0.82) [6]. No participants with LSS caused by tumor, trauma, or inflammatory conditions were included.

Assessment of chronic low back pain and disability

CLBP was defined as pain localized between the lower margin of the 12th rib and the inferior gluteal fold, persisting for more than three consecutive months. This definition was consistent with prior epidemiological studies in Japanese populations [23]. Presence of CLBP was determined through structured interviews during follow-up.

Functional disability due to CLBP was assessed using the Japanese version of the ODI, a well-validated tool that measures impairment across 10 domains: pain intensity, personal care, lifting, walking, sitting, standing, sleeping, social life, traveling, and sexual activity [24,25]. Each domain is scored from 0 to 5, and the total score is converted into a percentage scale (0-100%). Higher scores indicate greater disability.

DCLBP was defined as the presence of CLBP combined with an ODI score ≥21%, a threshold previously validated to reflect significant impairment in activities of daily living [25]. This threshold has been used in previous epidemiological and clinical studies to distinguish individuals with moderate to severe disability due to low back pain, and corresponds to functionally meaningful limitations in daily activities such as walking, sitting, and lifting [24,25]

Statistical analysis

All statistical analyses were performed using JMP version 16 software (SAS Inc., Cary, NC, USA). Baseline characteristics of participants with and without DCLBP were compared using the Student’s t-test for continuous variables and the chi-square test for categorical variables. Continuous variables were expressed as means ± standard deviation (SD), and categorical variables as counts and percentages.

To identify independent predictors of DCLBP, multivariate logistic regression analysis was conducted. The dependent variable was the presence of DCLBP at the seven-year follow-up, defined as the coexistence of CLBP and an ODI score ≥21%. The following baseline variables were included as explanatory variables based on their clinical relevance and prior research: age, sex, BMI, smoking status (current smoker vs. non-smoker), the MCS score of the SF-8, presence of severe lumbar spinal stenosis (grade 3 according to Suri’s classification), sum of SQ vertebral fracture grades at T11-L1, and the total lumbar DD score (sum of Pfirrmann grades from L1/2 to L5/S1).

Multicollinearity among explanatory variables was examined using the variance inflation factor (VIF), with a threshold of <2.0 considered acceptable. Odds ratios (ORs) and 95% confidence intervals (CIs) were calculated. A two-tailed p-value <0.05 was considered statistically significant.

## Results

Study population and follow-up

A total of 663 community-dwelling participants (mean age: 62 ± 13 years; 219 men and 444 women) were enrolled in the baseline cohort. Among these, 653 participants (98.5%) completed the seven-year follow-up and provided valid data on the ODI. These individuals were included in the final analysis, while 10 participants (1.5%) were excluded due to missing ODI responses.

Prevalence of disabling chronic low back pain

DCLBP was defined as low back pain persisting for more than three months combined with an ODI score ≥21%. Based on this definition, the prevalence of DCLBP was 13.9% (91 of 653 participants) at the seven-year follow-up (Figure 1).

Baseline characteristics by DCLBP status

Baseline demographic and clinical characteristics stratified by DCLBP status are presented in Table 1. Participants with DCLBP were significantly older than those without (mean age: 69.6 ± 10.7 vs. 60.8 ± 12.4 years, p < 0.0001). Women were more prevalent in the DCLBP group (71 of 91; 78.0%) compared with the non-DCLBP group (372 of 562; 66.2%), a difference that reached statistical significance (p = 0.0231). Participants with DCLBP also had a significantly higher mean body mass index (24.3 ± 3.4 kg/m² vs. 23.3 ± 3.5 kg/m², p = 0.0125).

**Table 1:** Baseline demographic and clinical characteristics of participants stratified by disabling chronic low back pain (DCLBP) status. Values are presented as mean ± standard deviation (SD) for continuous variables and number (percentage) for categorical variables. p-values were calculated using the independent t-test for continuous variables and the chi-square test for categorical variables. A p-value of <0.05 was considered statistically significant. The corresponding test statistic values (t or χ²) are shown in the second-last column.

Variable	DCLBP (+)	DCLBP (–)	Test Statistic	p-value
Number of subjects	91 (13.9%)	562 (86.1%)	–	N.A.
Sex (male:female)	21:70	198:364	χ² = 5.14	0.0231
Age (years)	69.6 ± 10.7	60.8 ± 12.4	t = 6.26	<0.0001
BMI (kg/m²)	24.3 ± 3.4	23.3 ± 3.5	t = 2.49	0.0125
Smoking habit (%)	14 of 91 (15.4%)	64 of 562 (11.4%)	χ² = 1.09	0.2961

No significant differences were observed between groups in terms of current smoking status (13 of 91; 14.3% in the DCLBP group vs. 65 of 562; 11.6% in the non-DCLBP group, p = 0.5046) or mean MCS scores from the SF-8 health survey (48.6 ± 9.2 vs. 49.0 ± 8.7, p = 0.6681).

Degenerative MRI findings

MRI-based degenerative spinal changes at baseline were significantly more pronounced among individuals who later developed DCLBP. Grade 3 LSS was identified in 43.7% (39 of 91) of participants with DCLBP, compared to 22.8% (128 of 562) in the non-DCLBP group (p < 0.0001).

The mean cumulative vertebral fracture score at the thoracolumbar junction (T11-L1) was significantly higher in the DCLBP group (2.7 ± 1.7) than in the non-DCLBP group (1.7 ± 1.5; p < 0.0001). Similarly, the total lumbar disc degeneration score (L1/2 to L5/S1) was significantly elevated in the DCLBP group (19.1 ± 2.3) compared to the non-DCLBP group (17.5 ± 2.5; p < 0.0001).

Multivariate logistic regression analysis

Multivariate logistic regression analysis was conducted to identify independent risk factors for DCLBP (Table 2). Older age was associated with increased odds of DCLBP (odds ratio [OR]: 1.07 per year; 95% confidence interval [CI]: 1.03-1.10; p < 0.0001), as was female sex (OR: 3.69; 95% CI: 1.83-7.44; p < 0.0001), and higher BMI (OR: 1.11 per kg/m²; 95% CI: 1.02-1.20; p < 0.0001).

**Table 2:** Multivariate logistic regression analysis of risk factors for disabling chronic low back pain (DCLBP). Odds ratios (OR) and 95% confidence intervals (CI) are shown for each variable. p-values were derived from multivariate logistic regression analysis. A p-value of <0.05 was considered statistically significant. DD: disc degeneration, SQ: semi-quantitative, SF-8: Short Form-8 Health Survey, MCS: mental component summary

Variable	Odds Ratio (95% CI)	p-value
Age (+1 year)	1.07 (1.03–1.10)	<0.0001
Sex (female vs male)	3.69 (1.83–7.44)	<0.0001
BMI (+1 kg/m²)	1.11 (1.02–1.20)	<0.0001
Smoking habit (yes vs no)	2.11 (0.74–6.05)	0.1626
SF-8 MCS (+1 point)	1.0 (0.96–1.05)	0.8473
Lumbar spinal stenosis (Grade 3 vs 0–2)	1.63 (0.93–2.85)	0.0925
Sum of SQ grades at T11–L1 (+1 point)	1.32 (1.10–1.60)	0.0024
Sum of DD grades at L1/2–L5/S (+1 point)	1.14 (1.01–1.30)	0.0305

MRI findings also remained significant predictors: each one-point increase in the sum of vertebral fracture grades at T11-L1 was associated with a 32% higher odds of DCLBP (OR: 1.32; 95% CI: 1.10-1.60; p = 0.0024), and each one-point increase in the lumbar disc degeneration score was associated with a 14% increase in odds (OR: 1.14; 95% CI: 1.01-1.30; p = 0.0305).

In contrast, smoking status (OR: 2.11; 95% CI: 0.74-6.05; p = 0.1626) and SF-8 MCS score (OR: 1.00; 95% CI: 0.96-1.05; p = 0.8473) were not significantly associated with DCLBP. No multicollinearity was detected among explanatory variables.

Graphical summary of predictors

**Figure 2:**
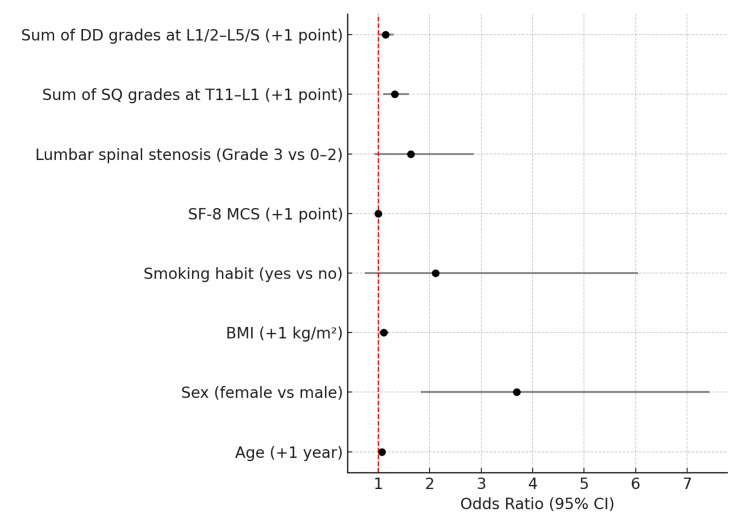
Forest plot of independent risk factors for disabling chronic low back pain (DCLBP). Multivariate logistic regression analysis was performed to identify predictors of DCLBP, defined as chronic low back pain persisting for more than three months with an Oswestry Disability Index (ODI) score ≥21%. The plot shows the adjusted odds ratios (ORs) and corresponding 95% confidence intervals (CIs) for each explanatory variable. Statistically significant predictors included older age, female sex, higher body mass index (BMI), greater sum of vertebral fracture grades at the thoracolumbar junction (T11–L1), and higher total lumbar disc degeneration grades (L1/2 to L5/S1). A red vertical dashed line denotes an OR of 1.0, indicating no association. Values are presented as ORs with 95% CIs. Statistical significance was defined as p<0.05. DD: disc degeneration, SQ: semi-quantitative, SF-8: Short Form-8 Health Survey, MCS: mental component summary

A forest plot summarizing the adjusted odds ratios and 95% confidence intervals for all variables included in the final regression model is provided in Figure 2. Predictor labels are clearly indicated to enhance standalone interpretability of the figure.

## Discussion

Summary of the results

In this seven-year longitudinal study of a general Japanese population, we identified lumbar DD, vertebral fractures at the thoracolumbar junction (T11-L1), and LSS as independent risk factors for DCLBP. Among the 653 participants who completed follow-up, 91 (13.9%) met the criteria for DCLBP, defined as chronic low back pain lasting more than three months and an ODI score of ≥21%. Multivariate analysis demonstrated that older age, female sex, higher BMI, higher sum of vertebral fracture grades at T11-L1, and greater lumbar DD severity were significantly associated with the risk of developing DCLBP. In contrast, smoking status and MCS scores from the SF-8 were not significant predictors.

Novel findings and strength of this study

This is the first population-based longitudinal study to evaluate lumbar DD, vertebral fractures, and LSS in combination as predictors of DCLBP over an extended follow-up period. While previous studies have demonstrated links between individual degenerative findings and LBP, this study uniquely assesses their simultaneous contributions to long-term functional disability using MRI-based assessments and a validated disability measure (ODI). Strengths of this study include its community-based sampling, the use of objective imaging data, and a clearly defined functional outcome, which enhance both internal validity and generalizability.

Comparison to previous studies

Our findings are consistent with earlier research identifying DD, vertebral fractures, and LSS as contributors to chronic LBP. Prior cross-sectional and clinical studies have shown that advanced DD (Pfirrmann grade 4 or 5) is associated with pain-related functional impairment [6,7]. Osteoporotic vertebral fractures are known to alter spinal alignment and biomechanics, leading to mechanical back pain and disability, particularly in older adults [8]. LSS has been widely recognized as a cause of neurogenic claudication and chronic discomfort due to nerve compression [9,10].

More recent evidence supports our findings. Udby et al. reported that Modic changes and reduced physical activity levels predicted long-term disability in a 13-year longitudinal study of LBP patients [26]. Young et al. found that LSS symptoms were common in individuals with knee or hip osteoarthritis, reflecting a broader musculoskeletal degenerative burden [27]. Hasserius et al. demonstrated that vertebral fractures are associated with long-term morbidity and mortality [28], while Carragee et al. emphasized the impact of psychosocial factors on LBP-related disability [29].

Our study expands upon this literature by evaluating these structural risk factors in combination and within a community-based longitudinal framework. The use of the ODI offers a more clinically meaningful endpoint than pain intensity alone, highlighting the progression from anatomical degeneration to real-life disability.

Relevant interpretation of the results

The association of older age, female sex, and higher BMI with DCLBP reflects well-established epidemiologic trends. Age-related degeneration, hormonal influences, and increased mechanical loading from higher BMI likely contribute to cumulative spinal stress and tissue failure [11]. Importantly, our study showed that the severity of degenerative findings, quantified by cumulative grades of vertebral fractures and disc degeneration, was more predictive of DCLBP than any single pathology alone.

The thoracolumbar junction (T11-L1) is biomechanically vulnerable due to its transitional location between the rigid thoracic spine and the more mobile lumbar segments. This region experiences high mechanical stress and is a common site for osteoporotic fractures [30]. Fractures here can alter sagittal alignment and lead to postural compensation, muscular strain, and chronic pain, thereby increasing the likelihood of persistent disability.

These findings underscore the need to assess spinal degeneration comprehensively, rather than in isolation, when evaluating risk for disabling outcomes.

Limitations

Several limitations should be noted. First, LBP and disability were self-reported, and while the ODI is validated, responses may be influenced by individual perception, pain threshold, and contextual factors. Second, this study focused on a Japanese cohort, and findings may not fully generalize to other populations with different ethnic, occupational, and health system contexts. Third, although our multivariate model adjusted for several important confounders, psychosocial variables such as depression, anxiety, and sleep disturbance were not included in the present analysis. However, these covariates were assessed in the broader ROAD study and could be explored in future analyses to enhance explanatory power.

Strengths of this study include its longitudinal cohort design with a seven-year follow-up period, the use of validated MRI and radiographic assessments to evaluate structural spinal changes, and the application of the ODI to quantitatively measure functional disability. Additionally, the use of a community-based sample enhances the generalizability of the findings to the general population.

## Conclusions

This study provides compelling longitudinal evidence that lumbar disc degeneration, vertebral fractures at T11-L1, and lumbar spinal stenosis are independent risk factors for disabling chronic low back pain in a general population. These findings emphasize the importance of comprehensive MRI-based spinal assessment in identifying individuals at elevated risk for disability.

From a translational standpoint, these results may inform early screening and public health strategies in super-aging societies, such as Japan, where proactive identification and intervention could mitigate long-term disability and healthcare burden. Future studies should incorporate genetic, psychosocial, and biomechanical factors to further elucidate the pathways from structural degeneration to chronic disability. Such efforts will support the development of multidisciplinary preventive strategies tailored to aging populations worldwide.
